# Preparing communities to receive persons recently suspected or diagnosed with COVID-19

**DOI:** 10.11604/pamj.supp.2020.35.2.23202

**Published:** 2020-05-05

**Authors:** Martin Lubega, John Emoit Ekol

**Affiliations:** 1 Soroti Regional Referral Hospital, Soroti District, Eastern Uganda; 2 Makerere University, Kampala, Uganda

**Keywords:** COVID-19, stigmatization, Uganda

## To the editors of the Pan African Medical Journal

Ever since the first case of COVID-19 was reported in Uganda, there are increasing number of reports of acts of discrimination and public stigmatization against rest travelers, people from areas affected by the epidemic, and those in quarantine. Unfortunately, this means that such people are being labelled, stereotyped, discriminated [1] and in worse situations mob justice has prevailed. It is so rejuvenating that some of our people that were recently diagnosed with COVID-19 are healing and are being discharged back home. This is a great milestone and spikes a ray of hope in our joint efforts to fight the pandemic in our country. However, there is a concern on how welcome these are into the communities. How prepared the families and communities are to receive such persons amidst the prevailing stigmatization of the disease in the public remains a very big question.

A case at hand is of a 30 year old female who returned from Kenya a few weeks back to her rented room in Kigandani village, Eastern Division in Soroti district. The surveillance team at Soroti regional referral hospital was notified of her return by the landlord and community leaders, she was picked up and put in the hospital quarantine for 14 days. In the due course, she was tested and found negative twice. Soon it was time for her to be discharged and return to the community. As a protocol here, the psychosocial team of the hospital surveillance and case management committee made a resettlement visit to the community to brief and prepare them to receive this lady prior to her discharge. From the interaction, community leaders, residents, and her landlord refused the team from discharging her back to their community in fear that she may still develop symptoms any time and infect them. They argued that she should be handed over to her family in her home village. Great resistance came from the landlord who insisted that other tenants were going to leave the rentals if this lady came back to the same rentals. It was such a trying moment for the hospital team led by the hospital´s principal mental health clinician that all their efforts to explain to the community were futile. At the end of it all the lady was discharged with another colleague from quarantine who was willing to stay with her in the meantime as she contacted relatives to pick her.

## Conclusion

This scenario sends signals of how much we need to continue sensitizing communities about COVID-19. It´s therefore of great importance that all hospitals handling suspects and those with the disease consider having a prior resettlement visit for every patient or suspect to prepare the community. This is important in preventing stigma that may be projected to such persons after discharge. Media houses should also continue sensitizing the public, at times its lack of information and misinformation that breeds and propagates such stigma. Stigma could potentially contribute to more severe health problems, ongoing transmission, and jeopardizes the efforts to control the outbreak [2]. We all have an important role to play in preventing and stopping stigma. We all need to be intentional and thoughtful of the information shared on social media and other communication platforms. There is need to ensure these are facts: stigma can be heightened by insufficient knowledge on how the disease is transmitted and treated, and how to prevent infection. Media reporting should therefore be balanced and contextualized to ensure dissemination of evidence-based information to help combat rumors and misinformation that could lead to stigmatization [2]. Gather information at regular intervals, from WHO website and Ministry of health platforms, in order to help you distinguish facts from rumors. Facts can help to minimize fears.

## Competing interests

The authors declare no competing interests.
